# Extended Loop Region of Hcp1 is Critical for the Assembly and Function of Type VI Secretion System in *Burkholderia pseudomallei*

**DOI:** 10.1038/srep08235

**Published:** 2015-02-04

**Authors:** Yan Ting Lim, Chacko Jobichen, Jocelyn Wong, Direk Limmathurotsakul, Shaowei Li, Yahua Chen, Manfred Raida, Nalini Srinivasan, Paul Anthony MacAry, J. Sivaraman, Yunn-Hwen Gan

**Affiliations:** 1 Department of Biochemistry, Yong Loo Lin School of Medicine, National University of Singapore, Singapore; 2 Department of Biological Sciences, National University of Singapore, Singapore; 3 Department of Microbiology, Yong Loo Lin School of Medicine, National University of Singapore, Singapore; 4 Department of Tropical Hygiene and Mahidol-Oxford Tropical Medicine Research Unit, Faculty of Tropical Medicine, Mahidol University, Bangkok, Thailand; 5 National Institute of Diagnostics and Vaccine Development in Infectious Diseases, School of Life Sciences, Xiamen University, Xiamen, China; 6 NUS Graduate School of Integrative Sciences and Engineering, National University of Singapore, Singapore; 7 Life Sciences Institute, Singapore Lipidomics Incubator, National University of Singapore, Singapore; 8 Life Sciences Institute, Immunology Program, National University of Singapore, Singapore

## Abstract

The Type VI Secretion System cluster 1 (T6SS1) is essential for the pathogenesis of *Burkholderia pseudomallei*, the causative agent of melioidosis, a disease endemic in the tropics. Inside host cells, *B. pseudomallei* escapes into the cytosol and through T6SS1, induces multinucleated giant cell (MNGC) formation that is thought to be important for bacterial cell to cell spread. The hemolysin-coregulated protein (Hcp) is both a T6SS substrate, as well as postulated to form part of the T6SS secretion tube. Our structural study reveals that Hcp1 forms hexameric rings similar to the other Hcp homologs but has an extended loop (Asp40-Arg56) that deviates significantly in position compared to other Hcp structures and may act as a key contact point between adjacent hexameric rings. When two residues within the loop were mutated, the mutant proteins were unable to stack as dodecamers, suggesting defective tube assembly. Moreover, infection with a bacterial mutant containing *in situ* substitution of these *hcp1* residues abolishes Hcp1 secretion inside infected cells and MNGC formation. We further show that Hcp has the ability to preferentially bind to the surface of antigen-presenting cells, which may contribute to its immunogenicity in inducing high titers of antibodies seen in melioidosis patients.

*B. *pseudomallei** is the causative agent of melioidosis, a disease characterized by a broad spectrum of clinical manifestations and a mortality rate of up to 40%^1^,^2^. The disease is endemic in Southeast Asia and Northern Australia^3^. It is a facultative intracellular bacterium that invades both phagocytic and non-phagocytic cells^4^,^5^. Internalized *B. pseudomallei* are capable of vacuolar escape into the cytoplasm, where the bacterium polymerizes actin on one pole to engage in actin-based motility^6^,^7^ and induces the formation of multinucleated giant cells (MNGC)^8^,^9^,^10^. Bacterial-induced MNGC formation requires bacterial motility^7^ and the Type VI Secretion System cluster 1 (T6SS1, also known as T6SS5), one of the six T6SSs *B. pseudomallei* possesses^11^,^12^.

T6SS has been reported to be responsible for mediating competitive or cooperative inter-bacterial interactions^13^,^14^. For *B. pseudomallei*, only T6SS1 is critical for bacterial pathogenesis *in* *vivo* in mammals^11^,^12^,^15^. In free-living bacteria, an AraC-type regulator BprC located within the adjacent Type III Secretion System cluster 3 (T3SS3) regulates basal T6SS1 expression^12^,^16^. However, T6SS1 expression increases by a 100 fold in infected host cells, and this requires VirAG, a two-component histidine kinase sensor–regulator system and to a lesser extent, BprC^12^. Within the host cells, VirAG is the major regulator of all T6SS1 genes beginning from *hcp*^12^. The only exception is the *tssAB* operon, which is regulated mainly by BprC^12^.

A core of 13 conserved genes encodes T6SS and their gene products form a macromolecular membrane-spanning syringe with a bacteriophage tail-like structure^17^. Hcp and VgrG (valine-glycine repeat protein G) are secreted substrates of T6SS that share homology with the bacteriophage tail and tail spike protein respectively^18^,^19^, but they are also required for T6SS function. Hcp is required for both the assembly of a functional T6SS and the export of T6SS substrates by chaperoning the substrates^20^, which includes itself. Crystallographic data of Hcp homologs from *Pseudomonas aeruginosa*, *Edwardsiella tarda*, enteroaggregative *Escherichia coli* and *Yersinia pestis* showed that the protein forms hexameric rings with an outer diameter of 80 Å and an internal diameter of 40 Å^21^,^22^,^23^,^24^. These data suggest that the hexameric rings could stack into tubes, which has been observed under crystallization conditions. Further evidence suggesting that Hcp forms tubes was obtained *in vitro* by the introduction of inter-hexameric disulfide bonds and through cross-linking^25^,^26^.

In this study, we have identified an extended loop region of Hcp1 (Loop L2, 3) that shows a significant movement away from the main Hcp β-barrel domain as compared to other known Hcp structures. We show that mutation in the loop affects the stacking of hexameric Hcp1, as well as abrogates secretion and the formation of MNGC during infection. This demonstrates how the extended loop region of Hcp1 from *B. pseudomallei* contributes to the structure and function of T6SS1 during infection. We also describe a novel property of Hcp1 where it preferentially binds antigen-presenting cells independent of the mutation in the loop region.

## Results

### The structure of *B. pseudomallei* Hcp1

**Figure 1:**
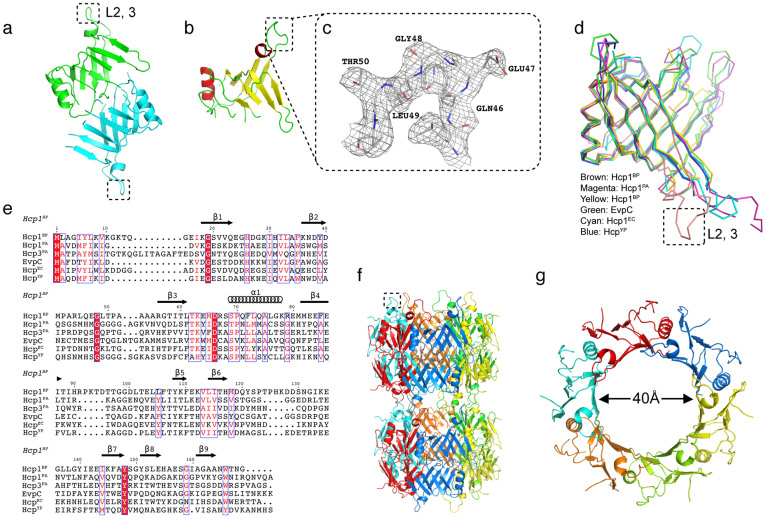
Crystal structure of Hcp1^BP^. (a) Asymmetric unit of Hcp1^BP^. Hcp1^BP^ molecule consists of residues from Ala3 to Asn168. The two monomers of Hcp1^BP^ are shown in green (monomer A) and cyan (monomer B). (b) Hcp1^BP^ monomer. α-helix (red), β-barrel (yellow), extended loop (Loop L2, 3) (demarcated with black dotted lines). (c) Final 2Fo-Fc electron density map (contoured at 1 σ) for the region Gln46 to Thr50. (d) The Cα superposition of Hcp1^BP^ with the structural homologs (Hcp1^PA^, Hcp3^PA^, EvpC, Hcp^YP^ and Hcp^EC^). (e) Sequence alignment of Hcp1^BP^ with other known structural homologs. (f) Stacking of Hcp1^BP^ generated by symmetry related molecules, which forms a tube-like architecture. (g) Hexameric ring of Hcp1^BP^. This ring has an outer diameter of 80 Å and an inner diameter of 40 Å. The extended loop (Loop L2, 3) is demarcated with black dotted lines in Fig 1a, Fig 1d and Fig 1f. Structure figures are prepared using PyMol.

So far, the crystal structure of Hcp homologs from *P. aeruginosa*, enteroaggregative *E. coli,* *E. tarda* and *Y. pestis* had been solved^21^,^22^,^23^,^24^. To understand the structure and function relationship of *B. pseudomallei* Hcp1, the crystal structure of recombinant Hcp1 was determined using the single wavelength anomalous diffraction method (SAD) with two molecules in the asymmetric unit (Fig. 1a). The model was refined to a final R-value of 0.248 (R_free_ = 0.285) up to 2.7 Å resolution and has good stereo-chemical parameters (Table 1). The Hcp1 molecule consists of residues from Ala3 to Asn168 (Fig. 1b). The region between residues Val21 to His31, His90 to Leu104 and Thr124 to Tyr140 were not defined in the electron density and hence not included in the model. Each molecule of Hcp1 consists of a β-barrel domain, with several loops predominantly on one end of the β-barrel and an α-helix (Ser69 – Lys78) located on one side of the β-barrel.

Notably, residues Asp40 to Arg56 form an extended loop (Loop L2, 3) that protrudes away from the β-barrel and is well defined in the electron density map (Fig. 1c). A search for topologically similar proteins within the Protein Data Bank (www.pdb.org) with the program DALI^27^ revealed significant structural homology between Hcp1^PA^ (PDB code 1Y12; rmsd = 3.3 Å for 105 Cα atoms; 18% sequence identity), Hcp3^PA^ (PDB code 3HE1; rmsd = 1.7 Å for 99 Cα atoms; 24% sequence identity), EvpC (PDB code 3EAA; rmsd = 3.3 Å for 105 Cα atoms; 23% sequence identity), Hcp1^EC^ (PDB code 4HKH; rmsd = 1.5 Å for 95 Cα atoms; 21% sequence identity) and Hcp^YP^ (PDB code 3V4H; rmsd = 1.6 Å for 90 Cα atoms; 21% sequence identity). A common feature of these molecules is the presence of an overall β-barrel architecture (Fig. 1d). However, significant differences were observed among these structural homologs in the loop regions. In particular, the major extended loop (Loop L2, 3) between Asp40 and Arg56 of Hcp1^BP^ is significantly shifted by 18–20 Å relative to its homologs. Furthermore, several loops of Hcp3^PA^ are also different in length and conformation, compared to Hcp1^BP^ (Fig. 1d). Although there are 10 amino acids conserved in the β-barrel domain (Fig. 1e), there is little or no conservation in the extended loop region (Asp40-Arg56) except for the following few residues: Gly48 in all the six proteins compared here, Gln46 in Hcp1^BP^, EvpC and Hcp3^PA^, and Pro42 in Hcp1^BP^, EvpC and Hcp^EC^.

In the crystals, the asymmetric unit consists of two Hcp1 molecules and the hexameric rings could be generated by a 6-fold symmetry with 12 molecules forming a two ring assembly (Fig. 1f). This stacking of hexameric rings could form the tube-like assembly with an outer diameter of 80 Å and an inner diameter of 40 Å (Fig. 1g). A similar hexameric ring stacking which forms a nanotube was previously reported^21^,^22^,^25^,^26^.

### Key residues in the hexameric interface

**Figure 2:**
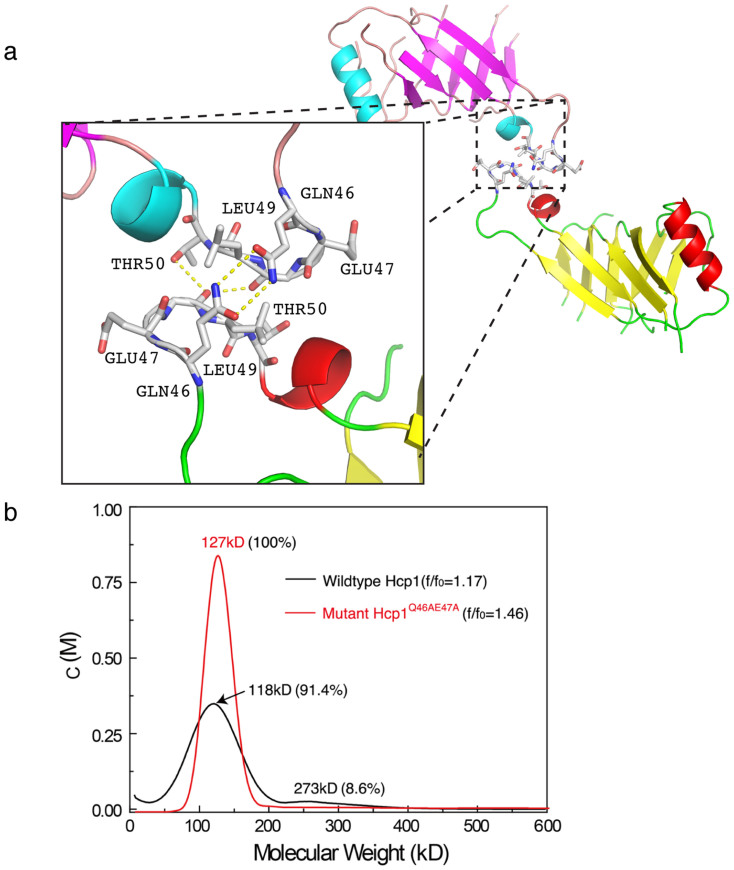
Putative critical inter-hexameric residues. (a) The interface between monomer A (pink and blue) and monomer B (yellow and red) is magnified in the insert. Residues with less than 3.5 Å are labelled (Glu47, Gln46, Leu49 and Thr50) and representative inter-residue hydrogen bonds are illustrated (yellow dotted lines). (b) Analytical ultra-centrifugation profile of wildtype (black histogram) and mutant Hcp1^Q46AE47A^ (red histogram) at 2 mg/mL concentration. The molecular weight kD refers to experimental mass.

The symmetry related molecules of the hexameric ring show that the extended loop region of Hcp1 could potentially act as a key contact point between two hexameric rings. There are at least 15 contacts (<3.5 Å) between the interacting monomers of the stacked hexameric ring. Most of them are hydrogen bond contacts involving the residues Gln46 to Thr50 from both interacting monomers of the stacked hexameric rings (Fig. 2a). These possible interactions prompted us to verify the role of the interacting loops in the stacking of the hexameric rings. We mutated glutamine and glutamic acid at positions 46 and 47 in this extended loop to alanines to generate Hcp1^Q46AE47A^. The effect of the Q46AE47A mutation in the oligomerisation of Hcp1 was investigated using analytical ultra-centrifugation (AUC) and dynamic light scattering (DLS). AUC analysis showed that at 2 mg/mL, wildtype Hcp1 demonstrated two peaks corresponding to hexamers and dodecamers (Fig. 2b, black histogram) whereas Hcp1^Q46AE47A^ exhibited a single peak that corresponded to hexamers (Fig. 2b, red histogram). The DLS experiment was in agreement with this observation albeit at a higher protein concentration (Supplementary Table S1). At 8 mg/mL, wildtype Hcp1 exhibited an apparent molecular weight of a dodecamer (equivalent to the stacking of two hexamers), whereas Hcp1^Q46AE47A^ mutant existed as a hexamer in solution. These results indicate that the mutated residues have a role in the stacking assembly of Hcp1.

### Point mutations in Hcp1 abolish MNGC formation without affecting protein expression

**Figure 3:**
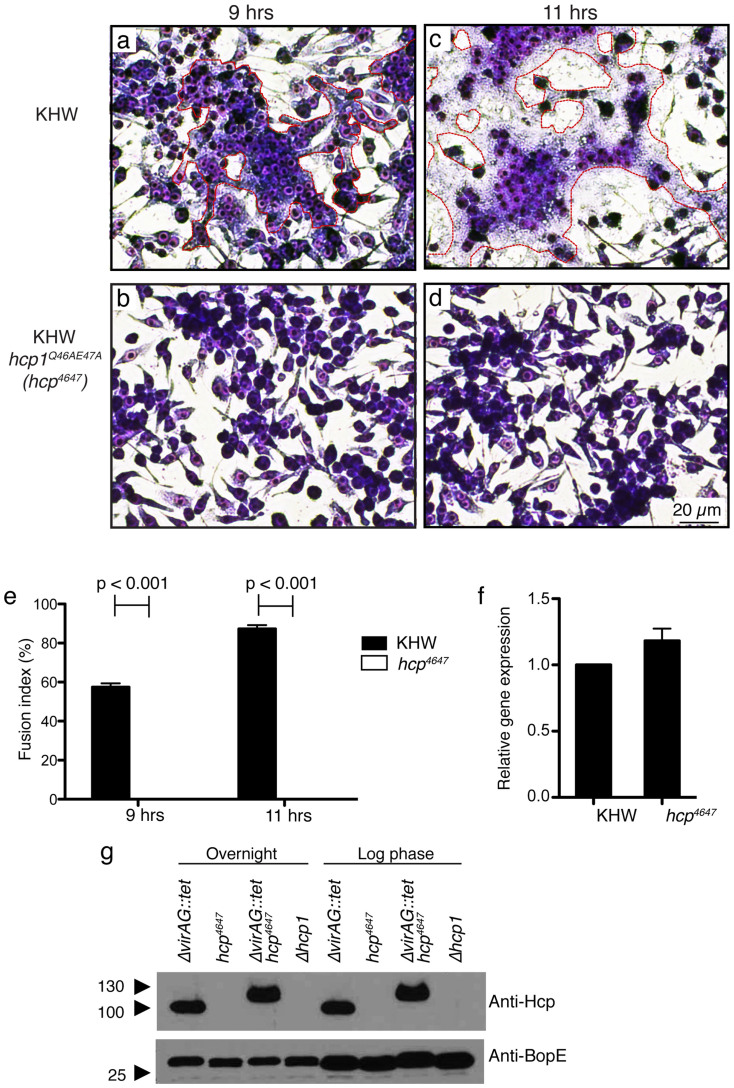
Effect of *in situ* Q46AE47A substitution on the function of Hcp1. RAW 264.7 macrophages infected with either overnight culture of wildtype KHW or KHW *hcp1*^Q46AE47A^ mutant were stained for MNGC formation at 9 h (a–b) or 11 h (c–d) post infection. (e) Transcript levels of *hcp1* in mutant *B. pseudomallei* during infection of RAW 264.7 cells were measured by real-time PCR and normalized to cells infected with wildtype bacteria. The *rpoB* gene is used as a housekeeping gene control. (f) Expression of Hcp1^Q46AE47A^ protein in *B. pseudomallei*. (g) Lysates from *ΔvirAG::tet*, KHW *hcp1*^Q46AE47A^, *ΔvirAG::tet* KHW *hcp1*^Q46AE47A^ and *Δhcp1* were resolved and immunoblotted for Hcp (Anti-Hcp) and BopE (Anti-BopE).

To determine how these mutations would compromise bacterial T6SS function, we introduced these mutations into the endogenous bacterial Hcp. We removed *hcp1* in wildtype *B. pseudomallei* and inserted a copy of *hcp1* with alanine substitutions at positions 46Q and 47E at the original chromosomal site via homologous recombination. We infected RAW264.7 cells with wildtype or the double substitution mutant KHW *hcp1*^Q46AE47A^ at 9 h (Fig. 3a and 3b) or 11 h (Fig. 3c and 3d). The double substitution mutant was unable to form MNGC at the time-points examined (Fig. 3e).

To exclude the possibility that the lack of MNGC formation was due to the mutation affecting gene transcription, we found that mRNA expression of *hcp1*^Q46AE47A^ was similar to wildtype KHW in infected RAW264.7 cells (Fig. 3f). The housekeeping gene *rpoB* was also expressed at similar levels, indicating that bacterial numbers were similar between wildtype and mutant bacteria infected cells. We had previously found that during the creation of the unmarked *ΔvirAG* deletion mutant, the intermediate mutant *ΔvirAG::tet*, where the *tet* cassette had not yet been flipped out, overexpressed the Hcp1 protein in the bacterial lysate (Supplementary Fig. S1). However, we were unable to detect secreted Hcp1 in bacterial culture supernatant from both wildtype and the mutant bacteria (data not shown). This could be due to low levels of secreted Hcp1 protein in this system and the loss of binding by our conformational-dependent antibody for the Hcp1 protein that had likely been denatured by the trichloroacetic acid (TCA)-precipitation from the supernatant. To determine whether the *hcp1*^Q46AE47A^ substitution mutant retained its ability to express the Hcp1 protein, the *ΔvirAG::tet* mutant had its endogenous *hcp1* replaced with *hcp1*^Q46AE47A^ *in situ*. The theoretical isoelectric points (pI) of the wildtype and mutant proteins are 5.68 and 5.86 respectively, hence the wildtype protein would migrate faster than the mutant protein under native conditions. We verified Hcp1 in the mutant was still produced in the bacterial lysate in the same amount as the KHW *ΔvirAG::tet* mutant with the original *hcp1* gene (Fig. 3g). Both of the *ΔvirAG::tet* mutant strains had been destroyed and no longer exist.

We reasoned that our conformational-dependent antibody might detect Hcp1 secretion during bacterial infection of macrophages when T6SS1 is highly expressed. We infected U937 cells with either wildtype or the mutant *hcp1*^Q46AE47A^ bacteria and stained the fixed cells with anti-Hcp1 and anti-lipopolysaccharide (LPS) antibodies, 4′,6-diamino-2-phenylindole (DAPI) to visualize the nuclei and wheat germ agglutinin (WGA) to delineate the plasma membranes. The staining was performed without permeabilisation because saponin interfered with the binding of the anti-Hcp1 antibody (Supplementary Fig. S2).

**Figure 4:**
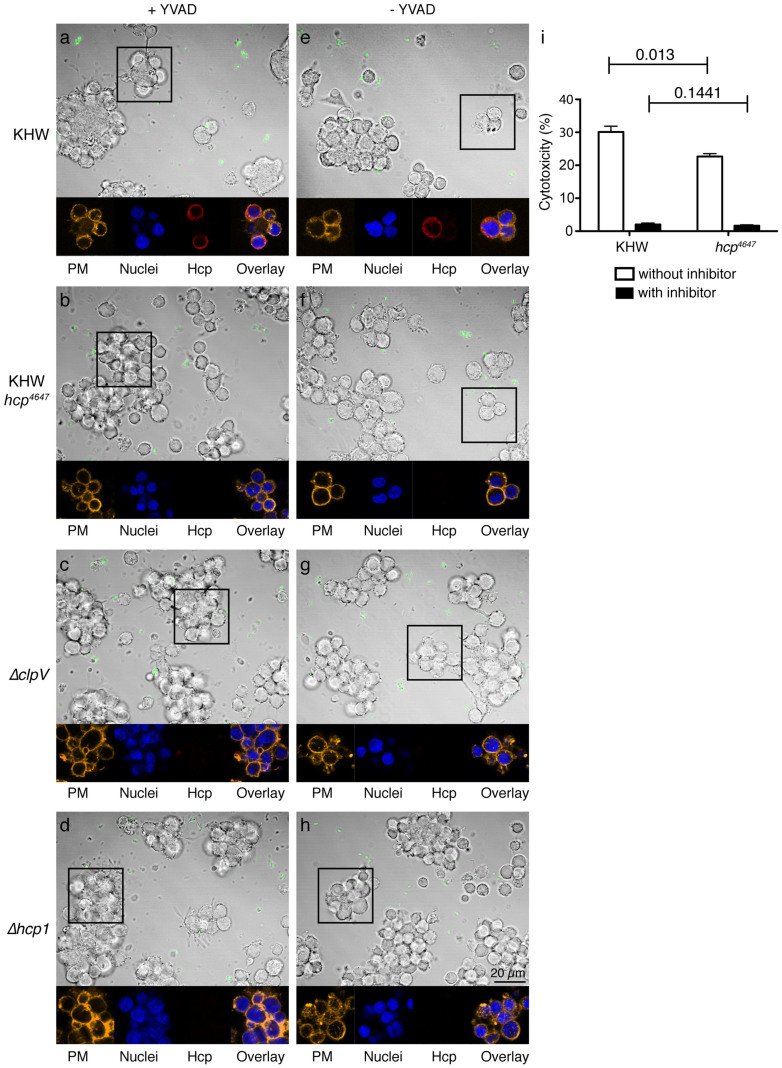
Secretion of Hcp1 protein during infection of U937 cells. PMA-activated U937s were infected with log phase wildtype bacteria KHW (a, e), KHW *hcp1*^Q46AE47A^ (*hcp*^4647^) mutant (b, f), *ΔclpV* (c, g), and *Δhcp1* (d, h). Boxes indicate the regions of interest for cells treated with (a–d) without YVAD (e–h). Mammalian cell nuclei were stained using DAPI (blue). Bacteria were stained using a rabbit polyclonal anti-LPS antibody and a secondary antibody AF488-conjugated anti-rabbit IgG (green). Cells were stained for Hcp1 using a mouse monoclonal antibody against Hcp1 and a secondary antibody AF647-conjugated anti-mouse IgG (red). The mammalian cell membrane was stained using AF555-conjugated WGA (yellow). Results shown are representative of two independent experiments. PMA (phorbol 12-myristate 13-acetate), DAPI (4′,6-diamindino-2-phenylindole), WGA (wheat germ agglutinin), PM (plasma membrane). (i) Cytotoxicity of U937 cells infected with wildtype or KHW *hcp1*^Q46AE47A^, treated with or without YVAD as measured by percentage of LDH release.

In some cells infected with wildtype bacteria, a ring-like pattern was observed for the Hcp stain (red), which did not co-localize exactly with the plasma membrane (yellow) (Fig. 4a and 4e). However, cells infected with the mutant did not show much Hcp staining (Fig. 4b and 4f), even though the overexpressed mutated protein was detected in the bacterial lysate (Fig. 3f). There were also little Hcp staining in the *ΔclpV*-infected cells (Fig. 4c and 4g), a mutant that was Hcp-secretion defective, and *Δhcp1*-infected cells (Fig. 4d and 4h). The DIC images were superimposed with staining for the extracellular bacteria (green). As *B. pseudomallei* infection of myeloid cells results in caspase-1-dependent cell death^28^, we treated the cells with the caspase-1 inhibitor YVAD to prevent cell lysis (Fig. 4i). There was no difference in the Hcp staining in cells treated with or without YVAD, showing that the differential Hcp detection between wildtype and mutant infected cells was not due to differential rate of cell lysis and release of bacterial proteins extracellularly. The presence of the bacterial protein in or on the macrophages provides evidence of the secretion of Hcp1 by the intracellular bacteria. We also confirmed that all four strains of bacteria (wildtype, *ΔclpV*, *Δhcp1* and *hcp1*^Q46AE47A^) were able to infect U937 cells to similar extent at the time-point examined by staining for intracellular bacteria (Supplementary Fig. S3). Thus, the lack of MNGC formation in the mutant *hcp1*^Q46AE47A^ bacterial-infected cells is most likely due to a secretion defect brought on by defective assembly, leading to a loss in T6SS1 function.

### Recombinant wildtype and mutant Hcp1 preferentially bind to antigen-presenting cells

**Figure 5:**
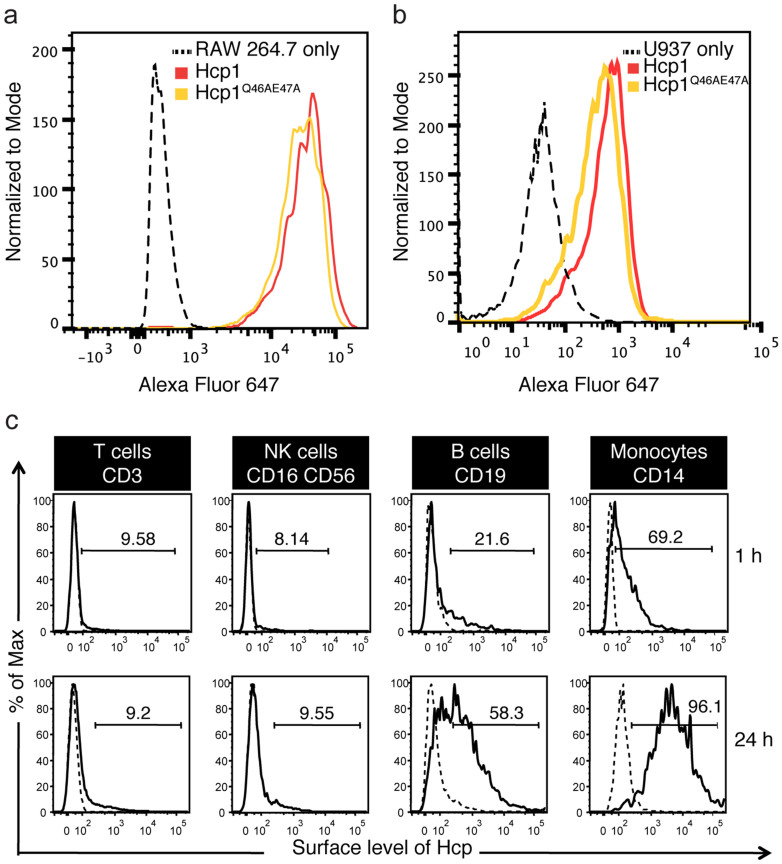
Affinity of Hcp1 for monocytic cell lines and antigen-presenting cells. 10 μg of Hcp1 was incubated with RAW 264.7 cells for 24 h (a), U937 cells for 24 h (b) or peripheral blood mononuclear cells for an hour or 24 h (c). They were stained either with (solid line) or without (dotted line) anti-Hcp antibody, followed by anti-mouse IgG AF647 secondary antibody.

The recombinant Hcp protein of *Aeromonas hydrophilia* had been reported to bind to macrophages and induced IL-10 and TGF-β^29^. We compared the ability of recombinant wildtype and mutant Hcp1 to bind murine macrophage cell-line RAW264.7 (Fig. 5a) as well as human monocytic cell-line U937 (Fig. 5b). Both wildtype and mutant proteins had comparable binding to the cells. To validate the association of Hcp1 with host primary cells, recombinant Hcp1 was incubated with peripheral blood mononuclear cells and found to preferentially bind antigen-presenting cells, namely B cells and monocytes (Fig. 5c). Surface-bound Hcp1 was detectable after one hour of incubation; with maximal binding at 24 h. Binding of Hcp1 to HEK293T was also observed, albeit to a lesser extent as compared to the B cells and monocytes (Supplementary Fig. S4). However, we could not detect any cell-signaling events triggered by the binding when we examined cell death, NF-κB and caspase-1 activation (Supplementary Fig. S5).

### Hcp1 is immunogenic in melioidosis patients

**Figure 6:**
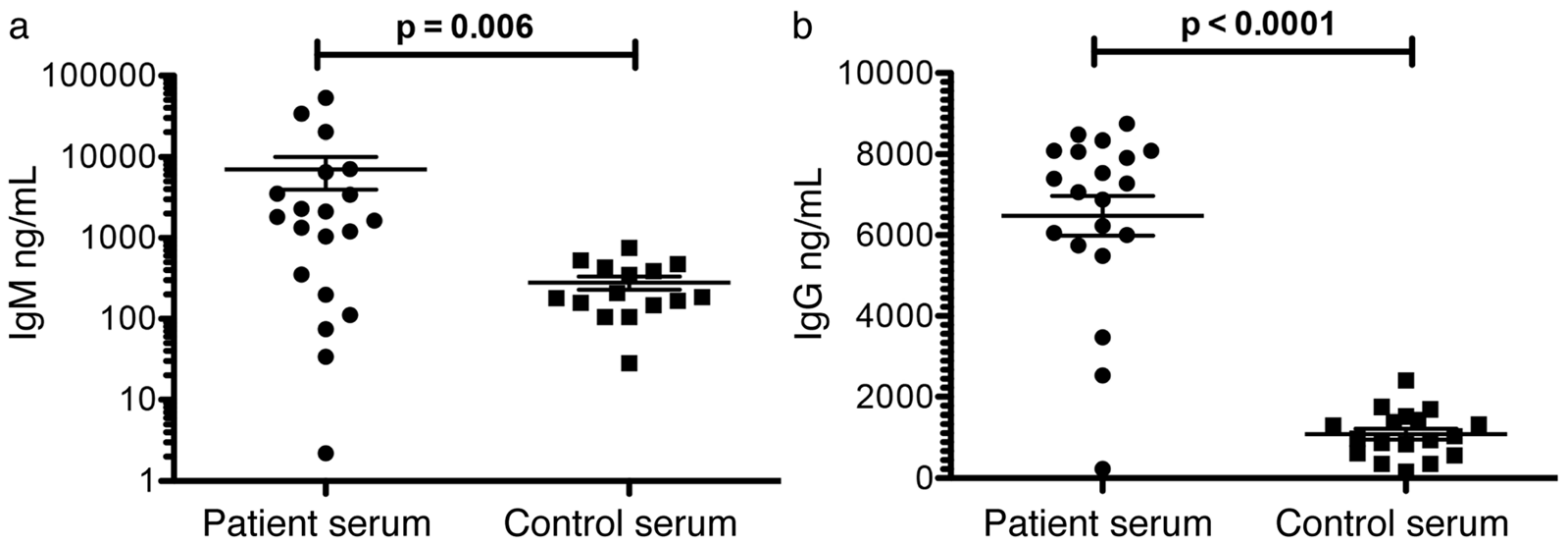
Serum antibody responses of patients with melioidosis to recombinant Hcp. Immunoglobulin G (IgG) (a) and Immunoglobulin M (IgM) (b) responses against native Hcp1 were assessed by ELISA in the serum of melioidosis patients or healthy individuals. Results are expressed as ng/mL of IgG and IgM.

The binding of Hcp1 to the surface of antigen-presenting cells could potentially increase its availability for antigen presentation to enhance immunogenicity during infection. To address whether Hcp1 is detectable during infection in humans, we examined the presence of the Hcp1 protein in sera of melioidosis patients (n = 20) versus healthy controls (n = 20) but were unable to detect it in both groups (Supplementary Fig. S6). However, we found the titers of anti-Hcp IgM and IgG in patients' sera to be significantly higher relative to healthy controls' (p = 0.006 and p < 0.0001 respectively) (Fig. 6a and 6b, respectively).

## Discussion

Studies have demonstrated that the Hcp protein is essential for both the assembly of T6SS and the export of its effectors^14^,^21^,^25^,^30^. We compared the crystal structure of *B. pseudomallei* Hcp1 with other available structures to predict a loop region that potentially contributes to stacking of the hexameric rings and therefore tube assembly. Through site directed mutagenesis of the loop region, we show that two charged residues in the loop region contribute to stacking of the hexameric rings *in vitro*. Through the creation of an *hcp* substitution mutant, we show that Hcp secretion *in vivo* during macrophage infection is affected and T6SS1 mediated MNGC formation in infected cells is abrogated. This finding helps substantiate the claim that tubular arrangement of Hcp rings contributes to T6SS function in a physiologically relevant context.

Up to now, Hcp tube assembly, as defined by Hcp hexamer stacking, has been engineered through cross-linking and mutations in cysteine residues *in vitro*^25^,^31^. Douzi *et al*^24^ reported that the K_D_ of the intrahexameric interactions of Hcp is 7.2 μM, which is weak. This could be the reason why native proteins of Hcp have not been observed to form tubes spontaneously *in vitro*. Furthermore, Brunet *et al* showed that introduction of bulky residues into the hexamer interface resulted in a loss of T6SS secretion^25^. A unique aspect of *B. pseudomallei* Hcp1 protein is the significant deviation of its extended loop region (Loop L2, 3) from other Hcp homologs. The residues Q46 and E47 located at the tip of the extended loop of the β-barrel core structure of Hcp1 forms hydrogen bonds with the residues from the adjacent Hcp1 molecule in the stacked rings generated by the symmetry related molecules. We thus speculated that these hydrophilic/charged residues play an important role in hexamer stacking through the electrostatic interactions and enable the formation of stable stacks. Our DLS and AUC data support this, as the mutated Hcp could no longer stack into dodecamers. If the bacterium carries these mutations, T6SS1 secretion and T6SS1 mediated cell fusion will theoretically be abolished due to defective T6SS assembly during bacterial infection of host cells. Our results support this hypothesis and demonstrate the importance and relevance of Hcp1 tube assembly in mediating T6SS1 function in the physiological context of a bacterial infection, when T6SS1 is highly induced. Whether the loop regions of other Hcp homologs are also involved in hexameric stacking remains to be explored.

The detection of endogenous Hcp in all bacterial cellular compartments and culture medium had been reported^22^,^32^,^33^. In *B. pseudomallei*, the overexpression of VirAG *in trans* is necessary for the detection of T6SS1 secreted substrates Hcp1 and VgrG in bacterial cultures^34^. This is because the expression of T6SS1 in free-living bacteria is very low^12^. Thus, to our knowledge, this is the first instance that the detection of endogenous Hcp during a bacterial infection had been imaged, allowing us to visualize the secretion of T6SS proteins in infected host cells. Hcp1 localizes to the plane of the plasma membrane of infected cells, but the signals did not co-localize with that of WGA. Perhaps the membrane receptors of Hcp1 and WGA do not overlap. It is unclear how Hcp1 localizes to the surface of the infected cells. It could be residing under the plasma membrane, or getting out of the cells (we have excluded cell lysis) and binding to the surface of the cells. In the mutant *hcp1*^46^,^47^ bacterial-infected cells, we did not detect Hcp staining even though the antibody detected the mutated protein in VirAG-overexpressed bacterial lysate, indicating that lack of detection is not due to a loss of epitope or loss of Hcp expression, but a defect in secretion.

Although *B. pseudomallei* T6SS1 is essential for bacterial pathogenesis *in vivo*^11^,^12^,^15^, the only defined *in vitro* function ascribed to *B. pseudomallei* T6SS1 is the formation of MNGC. There are also reports on its partial contribution to intracellular replication and cytotoxicity of host cells^11^,^12^, but it is unclear whether these are direct effects mediated by T6SS1 or are consequences of cell fusion. VgrG has been identified as a T6SS1 substrate through proteomic analysis of the *B. thailandensis* secretome via the overexpression of VirAG^35^ and the C terminal domain (CTD) of VgrG had been shown to be necessary for MNGC formation^35^,^36^. Its secretion is also dependent on Hcp expression^37^. Using MNGC formation as readout for T6SS1 function, we show that the inability to secrete Hcp (through the use of the *hcp* double substitution mutant) also prevents cell fusion. We believe this is strong evidence that the mutant Hcp is unable to support VgrG secretion and therefore, MNGC formation.

Hcp1 from *B. pseudomallei* had previously been shown to be recognized on an immunoblot by pooled sera of melioidosis patients^11^. The majority of our melioidosis cohort produced high titers of anti-Hcp1 IgG, indicating that Hcp1 is a significant target for the host humoral immune response. The ability of Hcp1 to bind to the surface of host cells, particularly its preference for antigen-presenting cells, could contribute to its immunogenicity by increasing uptake and antigen presentation to T helper cells, which are required for the generation of the antibody response and isotype switching. The fact that the mutant protein also binds similarly shows that hexameric stacking is not necessary for binding. A high anti-Hcp response was also previously reported in cystic fibrosis patients with chronic *P. aeruginosa* infections^21^, indicating that Hcp is available for immune processing during bacterial infection. However, it is unclear whether there is any physiological significance in terms of bacterial pathogenesis in Hcp1 binding to host cells. Unlike the report by Suarez *et al* where Hcp binding to cells suppressed the host immune response and facilitated bacterial replication and spread^29^, we could not find any effect of Hcp binding on cell signaling or bacterial pathogenesis. Hcp has recently been described to be a chaperone and co-receptor for T6SS substrates^20^, indicating its ability to interact with other proteins. Perhaps the binding of host surface proteins occurred due to similarities between host surface proteins and its natural substrates.

In conclusion, we have shown that mutations in the extended loop region of *B. pseudomallei* Hcp1 disrupt the stacking of the hexameric rings and the formation of the tube-like assembly. Therefore, our cell infection model complements the protein structural studies, demonstrating that stacking of the hexamers and tube assembly is essential for secretion and function during bacterial infection.

## Methods

Reagents are from Thermo Scientific and antibodies are from BD Pharmingen unless specified otherwise.

### Cloning and generation of recombinant proteins

The *hcp1 gene* from *B. pseudomallei* was cloned in frame with the C-terminus His-tag of the pET-22b plasmid (Merck KGaA) and transformed into BL21 (DE3). Recombinant Hcp was purified using B-PER 6xHis Fusion Protein Purification Kit. Site-specific mutations in Hcp1 were introduced by overlapping PCR as previously described^38^ and verified by DNA sequencing.

### Purification, crystallization and structure determination

Transformed *E. coli* BL21 cells were grown in defined M9 medium supplemented with 25 mg/L L-SeMet at 37°C until O.D_600 nm_ reached 0.6. Cells were induced with 100 μM IPTG at 37°C for 5 h and lysed in 40 mL of lysis buffer (50 mM Tris-HCl [pH 7.0], 0.2 M NaCl, 1% (w/v) Triton X-100, 5% (w/v) glycerol, 5 mM DTT, and protease inhibitors (Roche). The protein was purified by affinity and size exclusion chromatography (Superdex 75, GE Healthcare). Drops containing 1 μL of protein solution (8 mg/ml) and 1 μL of reservoir solution were equilibrated by hanging drop vapor diffusion at 25°C. Crystals were grown from 0.1 M Na HEPES 7.5 1.4 M tri-sodium citrate (with the protein in 20 mM Tris-HCl (pH 7.0), 200 mM NaCl and 5% (w/v) glycerol and were cryoprotected in the reservoir solution supplemented with 25% glycerol and flash cooled at 100 K. The structure was determined using SeMet-labeled protein crystals via the SAD method^39^. X-ray diffraction data were collected at beamline 13B, National Synchrotron Radiation Research Centre (Taiwan) using a Quantum-315r CCD area detector (ADSC) and processed with HKL2000^40^. Phasing was carried out using Phenix-Autosol^41^ followed by model building with RESOLVE^42^. The remaining residues were manually built using COOT^43^, followed by refinement using phenix-refine^41^. During the refinement, an analysis using the phenix-Xtriage program indicated the presence of merohedral twinning. This was continued with the twin refinement until the R-value converged to 0.248 (R_free_ = 0.285) for reflections I > σ (I) to 2.7 Å resolution (Table 1).

### Analytical Ultra-Centrifugation

The oligomeric state of Hcp1 was investigated by monitoring its sedimentation properties in sedimentation velocity experiments, using 400 μL of samples at 2.0 mg/ml in PBS buffer. Sedimentation velocity profiles were collected by monitoring the absorbance at 280 nm. Samples were centrifuged at 40,000 rpm at 20°C in a Beckman Optima XL-I centrifuge fitted with a four-hole AN-60 rotor and double-sector aluminum centerpieces and equipped with absorbance optics. The scans were analysed using Sedfit program^44^.

### Bacterial mutant generation

Methods are described in supplementary methods. All bacterial strains used are listed in Table 2.

### Giemsa stain for MNGC formation

2.5 × 10^5^ RAW 264.7 cells were seeded and infected with wildtype *B. pseudomallei* or *hcp1*^Q46AE37A^ mutant at an multiplicity of infection (MOI) of 10:1. Kanamycin (250 μg/mL per well) was added an hour post-infection and infected cells were stained with Giemsa at specific time-points post-infection. The fusion index was estimated as previously described with modifications^8^,^45^. Each well was divided into four quadrants. Cells located in the middle of the field were omitted because they were too dense to enumerate. Images from each quadrant were taken using 10× magnification. The total number of nuclei within MNGCs (>3 nuclei/cell) and the total number of cells from each quadrant was counted, and summated from all four quadrants. The fusion index is the ratio of the total number of nuclei within MNGCs to the total number of cells multiplied by 100%.

### Cell infection and measurement of gene expression by real-time PCR

5 × 10^5^ cells per well of RAW 264.7 cells were infected with overnight culture wildtype *B. pseudomallei* or *hcp1*^Q46AE37A^ mutant at an MOI of 10:1, for 9 h, with 250 μg/mL of kanamycin added an hour post-infection. RNA were isolated from infected RAW 264.7 using PureZol (Bio-Rad) and illustra RNAspin Mini kit (GE Healthcare). cDNA was synthesized using RevertAid First Strand cDNA Synthesis Kit. Transcripts were quantified using iQSYBR® Green Supermix for iCycler (Bio-Rad) in a Bio-Rad iQ5 machine, with *rpoB* as the reference gene. Results were reported as means with standard deviation of duplicate infection conditions per bacterial strain.

### Immunoblotting

Overnight or log phase culture of *ΔvirAG::tet*, KHW *hcp1*^Q46AE37A^ (*hcp4647), ΔvirAG::tet hcp1^Q46AE47A^* or *Δhcp1* were lysed in B-Per® supplemented with EDTA-free protease inhibitor cocktail (Roche) and filter sterilized. Lysates were concentrated (MWCO 5,000 Da) and resolved with 10% Native-PAGE or 12.5% SDS-PAGE gels. Proteins were transferred onto polyvinylidene fluoride membranes and probed with anti-Hcp antibody (clone 56-1) or anti-BopE rabbit polyclonal antibody^46^, followed by their respective secondary antibodies (goat anti-mouse and goat anti-rabbit) conjugated with HRP. Blots were developed using ECL plus reagent (GE Healthcare).

### Confocal microscopy

U937 cells (2.5 × 10^5^ cells per coverslip) were seeded onto coverslips and activated with phorbol 12-myristate 13-acetate (PMA) for 24 h, followed with fresh medium without PMA for a further 24 h. Cells were treated with 40 μM of Ac-YVAD-cmk an hour prior to and during the infection. Cells were infected with log phase KHW, KHW *hcp1*^Q46AE47A^, *ΔclpV* and *Δhcp1* at a MOI of 20:1. Kanamycin (250 μg/mL per well) was added an hour post infection and cells were fixed with 1% paraformaldehyde (PFA) after a further infection of 6.5 h. Supernatants were harvested. Cells were washed with PBS-10 mM glycine and stained with anti-Hcp antibody clone 56-2-2 and a rabbit anti-*B. pseudomallei* LPS antibody, both at 1:1000 dilution for an hour. Cells were subsequently stained with DAPI at 1:1000 dilution, goat anti-mouse Fab' conjugated with Alexa Fluor® 647 (AF647) and goat anti-rabbit Fab' conjugated with Alexa Fluor® 488 (AF488) at the dilution of 1:500, followed by WGA conjugated with Alexa Fluor® 555 (AF555) at the dilution of 1:000. The coverslips were mounted using ProLong® Gold Antifade reagent. Images were acquired using LSM 710 MicroImaging System (Carl Zeiss).

### LDH release assay

The harvested supernatants were assayed using the lactate dehydrogenase (LDH) kit (Clontech). Results are representative of three independent experiments and are expressed as means with standard deviation of triplicate infection conditions per bacteria strain.

### Flow cytometry

Human peripheral blood mononuclear cells (PBMCs) were isolated from peripheral blood by Ficoll gradient. U937, RAW 264.7 cells or PBMCs were incubated with Hcp1 (10 μg of protein per 5 × 10^5^ cells) for an hour or overnight with rotation at 37°C. Coated cells were washed in PBS and stained with anti-Hcp antibody (clone 56-1) at the dilution of 1:100, followed by goat anti-mouse AF647 at the dilution of 1:200. PBMCs were further stained with antibodies against either CD19-PE/CD20-FITC, CD14-PE, CD16-PE/CD56-APC, or CD3-eFluor®450 (eBioscience)/CD4-PE/CD8-FITC. Stainings were done at 4°C for 30 min, and cells were fixed with 1% PFA. Cells were analyzed by flow cytometry on a BD Fortessa FACS Scan.

### ELISA serum assay

MaxiSorp plates were coated with Hcp and blocked with 5% skim milk in PBS-T. Patients' and controls' sera were added at a dilution of 1:100. Wells were incubated with goat anti-human antibody conjugated with HRP at a 1:5000 dilution and developed with 3,3′,5,5′ tetramethylbenzidine (TMB) (BD Biosciences). The reaction was stopped with H_2_SO_4_ and plates were read at 450 nm. A standard curve was generated by coating human IgG or IgM in a 10-fold dilution series (10 pg/mL – 100 μg/mL). Patients' sera were from culture-confirmed melioidosis patients presenting at Sappasithiprasong Hospital^47^. The 20 patients were randomly selected from the patients recruited into the study. Control sera were from healthy consenting donors (age range 25–50).

### Statistical analysis

Statistical significance was performed using the Student's *t*-test except for Figures 6a and b (non-parametric, two-tailed Mann-Whitney test). Data were considered significant at a *p* value of ≤0.05.

### Ethical Statement

Ethical approval for melioidosis patients was obtained from the Ethics Committee, Faculty of Tropical Medicine Mahidol University^47^. Ethical approval for healthy controls was obtained from the National University of Singapore Institutional Review Board covering all work described with human blood-derived cells and antibodies, under the protocol number 07–101. Written informed consent was obtained from all study participants and the clinical investigation conducted according to the principles in the Declaration of Helsinki.

Ethical approval was obtained from the National University of Singapore Institutional Animal Care and Use Committee covering all work described with animals, under the protocol number 048/08 and in accordance with the National Advisory Committee for Laboratory Animal Research (NACLAR) Guidelines (Guidelines on the Care and Use of Animals for Scientific Purposes). NUS is an AAALAC accredited institution.

## Additional Information

**Accession codes** Coordinates of Hcp1 have been deposited in the Protein Data Bank (http://www.pdb.org) under accession code 3WX6.

## Supplementary Material

Supplementary Information Supplementary information

## Figures and Tables

**Table 1:** Data collection, phasing and refinement statistics

Data collection		
Space group:	P6	
Cell Dimensions		
a,b,c (Å)	82.74 82.74 64.79	
α,β,γ (^0^)	90 90 120	
Resolution range (Å):	25–2.62 (2.71–2.62)	
R_Sym_^a^:	0.115 (0.274)	
Overall (I/σI):	15.4	
Completeness (%):	100 (100)	
Redundancy:	29.5 (29.2)	
**Refinement**		
Resolution range:	15–2.70	
Number of Reflections	7575	
R_work_^b^/R_free_^c^	0.248/0.285	
**Number of Atoms**		
Protein	1719	
Ligand	0	
Water	57	
**B-factors**		
Protein	31.5	
**R.M.S. deviations**		
Bond length (Å)	0.01	
Bond angles (^0^)	1.219	

Statistics from the current model.

^a^R_sym_ = Σ|I_i_ − <I>|/Σ|I_i_| where I_i_ is the intensity of the i^th^ measurement, and <I> is the mean intensity for that reflection.

^b^R_work_ = Σ| F_obs_ − F_calc_|/Σ|F_obs_| where F_calc_ and F_obs_ are the calculated and observed structure factor amplitudes, respectively.

^c^R_free_ = as for R_work_, but for 10.0% of the total reflections chosen at random and omitted from refinement.

*Values in the parenthesis are the highest resolution bin values.

**Table 2:** List of plasmids and strains

Plasmid or Strain	Relevant characteristic(s)^a^	Source or reference	
**Plasmid**			
pK18*mobsacB*	Conjugative, suicide vector, Km^r^	48	
pFRTT1	pGEM-T contains a *tet* cassette with FRT sites and *tetRA* genes, Ap^r^, Tc^r^	16	
pET28a	Protein expression vector with N-terminal His-tag/thrombin/T7-tag and optional C-terminal His-tag	Novagen	
pET22b	Protein expression vector with N-terminal His-tag and optional C-terminal His-tag	Novagen	
***B. pseudomallei***			
KHW	*B. pseudomallei* wildtype strain	49	
*Δhcp1*	KHW, codon 21–160 of BPSS1498 was removed	This study	
*ΔclpV*	KHW, codon 11–936 of BPSS1502 was removed	This study	
KHW *hcp1*^Q46AE47A^	KHW, codon 46 and 47 of BPSS1498 substituted with alanine	This study	
***E. coli***			
S17-1	Donor strain for conjugation	50	

^a^Abbreviations: Ap^r^, ampicillin resistant; Km^r^, kanamycin resistant; Tc^r^, tetracycline resistant, Tm^r^, trimethoprim resistant.
