# Supplementary material for: Extended Loop Region of Hcp1 is Critical for the Assembly and Function of Type VI Secretion System in Burkholderia pseudomallei
Source: Sci Rep. 2015 Feb 4;5:8235. doi: 10.1038/srep08235 (PMC4650826; doi:10.1038/srep08235)
Supplement: Supplementary Information [file srep08235-s1.pdf]

Supplementary Information

Extended Loop Region of Hcp1 is Critical for the Assembly and Function of Type VI Secretion System in *Burkholderia pseudomallei*

Yan Ting Lim, Chacko Jobichen, Jocelyn Wong, Direk Limmathurotsakul, Shaowei Li, Yahua Chen, Manfred Raida, Nalini Srinivasan, Paul Anthony MacAry, J. Sivaraman*, Yunn-Hwen Gan*

Supplementary Methods

Bacterial strains

Bacterial strains and plasmids used in this study are listed in Table 2, and primers are listed in Supplementary Table S2. Mutants created were derived from a clinical strain of *B. pseudomallei* KHW [38]. *B. pseudomallei* gene deletions and *in situ* site-directed alanine substitutions were generated by allelic exchange as described[12] using In-Fusion PCR cloning kit (Clontech) and cloned into pK18mocsacB. The plasmids were introduced into *B. pseudomallei* strains by conjugation with *E. coli* S17-1. The conjugated bacteria were counter-selected in LB + 15% sucrose and screened using colony PCR.

The bacteria strains KHW, *Δhcp1,* KHW *hcp1*^{Q46AE47A}*, ΔclpV, ΔvirAG::tet* and *ΔvirAG::tet hcp1*^{Q46AE47A} were cultured in Luria Broth (LB) media. For the overnight cultures, the strains were grown in 2 mL of LB. For the log phase cultures, their respective overnight cultures were diluted 1:20 in LB and cultured for 2 hrs till the approximate optical density (OD_{600}) of 0.5.

Cell culture

The cell lines U937 (human myelomonocytic lymphoma) and RAW 264.7 (murine leukaemic monocyte macrophage) were obtained from American Type Culture Collection. The infection media is R10 (RPMI 1640 (Invitrogen) supplemented with 10% heat-inactivated fetal bovine serum and 2mM L-glutamine (Invitrogen).

Generation of monoclonal antibody against Hcp1

Monoclonal antibodies against Hcp1 were generated as previously described.[51] Hybridoma supernatants were assayed for anti-Hcp response by ELISA, by flow cytometry using Hcp1-coated U937 cells or by confocal microscopy using infected U937 cells.

Staining intracellular bacteria in U937 cells

U937 cells were prepared, infected and stained as aforementioned in the Materials and Methods section with the following modifications: The fixed cells were permeabilized and stained in 0.2 % saponin in PBS-glycine with a rabbit anti-*B. pseudomallei* LPS antibody at the dilution of 1:1000 for an hour, followed by wheat germ agglutinin conjugated with AF555 at the dilution of 1:000 and a AF488-conjugated anti-rabbit IgG secondary antibody.

Binding of Hcp1 to HEK 293T cells.

HEK 293T cells were incubated with Hcp1 (30 μg of protein per 5 x 10^5 cells) for an hour or overnight with rotation at 37 ^{o}C. Coated cells were washed once in PBS and stained with anti-Hcp antibody at the dilution of 1:100, followed by goat anti-mouse AF647 at the dilution of 1:200, at 4 ^{o}C for 30 min per stain. The cells were fixed with 1% PFA and analyzed by flow cytometry on a BD Fortessa FACS Scan.

Hcp1 capture ELISA

Microtitre MaxiSorp plate was coated with polyclonal rabbit anti-rHcp1 antibody and blocked with 5 % skim milk in PBS-T. Patients' and controls' sera were diluted 1:2 and added to blocked plates at room temperature for 1 hr. Wells were washed and incubated with the mouse anti-rHcp1 antibody (56-1) per well for 1 hr and detected

with a goat anti-human secondary antibody conjugated with HRP at a dilution of 1:5000 per well in blocking buffer at room temperature for another hour. The wells were developed with TMB and the reaction was stopped with H_2SO_4. The wells were read at 450 nm (415 nm as the reference wavelength). A standard curve was generated by adding rHcp1 in a 2-fold dilution series of the range coating human IgG or IgM in a 10-fold dilution series of the range 62.5 pg/mL- 4000 pg/mL.

^{51}Cr release assay

Target U937 cells were pulsed with Chromium-51 (^{51}Cr) (PerkinElmer) for 1 hr at 37 ^{o}C. 1 x 10^6 of target cells were incubated either with 30 μg or 0.1 μg of rHcp1, for either 4 hr, 6 hr or overnight at 37 ^{o}C. The supernatants were centrifuged, harvested and 30 μL of supernatant per condition were added into a LumaPlate (PerkinElmer). The plates were air dried, sealed and counted with TopCount® (PerkinElmer).

NF-κB-SEAP reporter assay

0.5 x 10^6 THP1-Blue™ (InvivoGen) cells were stimulated with either 1 μg of lipoarabinomannan from *Mycobacterium tuberculosis*, 4 μg of endotoxin-free rHcp1 or BSA for 24 hr. The supernatants were harvested, and for each condition, 20 μL of supernatant was added to 20 μL of QUANTI-Blue substrate (InvivoGen). The reaction was incubated for 24 hr at 37 ^{o}C, and absorbance was read at 650 nm.

IL-1β assay

2 x 10^6 J774.1 murine macrophages were stimulated with either 10 μg of rHcp1, BSA or adenosine triphosphate (ATP), with or without LPS. They were stimulated either for 4 hr or overnight at 37 ^{o}C. The supernatants were harvested, the cell debris pelleted and removed, and concentrated with a 5,000 MWCO concentrator to 400 μL. IL-1β levels was measured in triplicates with the Human IL-1β ELISA MAX kit according to manufacturer's instructions (BioLegend).

Dynamic light scattering (DLS)

DLS measurements were performed at room temperature on a DynaPro (Protein Solutions) DLS instrument. The quality of the data is represented in the sum of squares (SOS) error statistic reported for each sample acquisition (a single correlation curve).

Supplementary Figures.

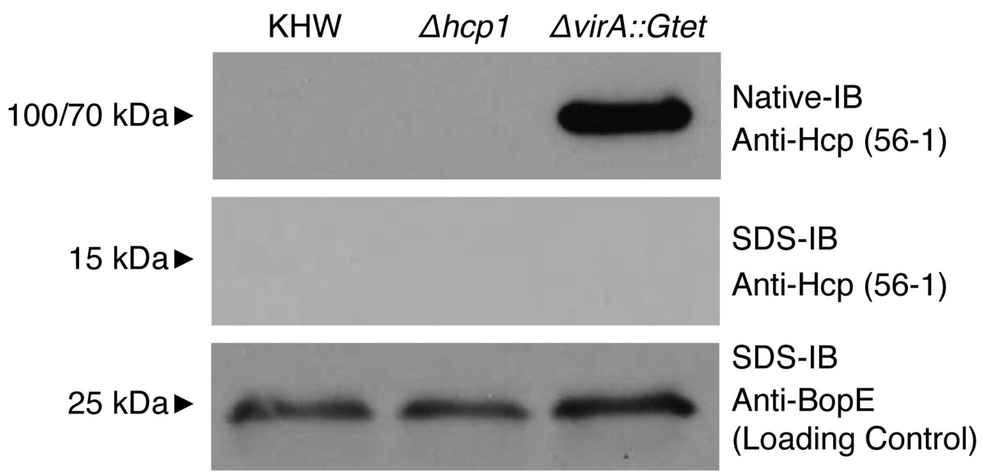

Figure S1: Specificity of 56-1 for native Hcp1. Lysates from KHW, *Δhcp1* and *ΔvirAG::tet* were resolved and immunoblotted in their native form (Native-IB) or denatured form (SDS-IB). The expected size of native hexameric Hcp1 is approximately 108 kDa, and the denatured monomer 18 kDa. The loading control is BopE, a 25 kDa protein belonging to the T3SS3.

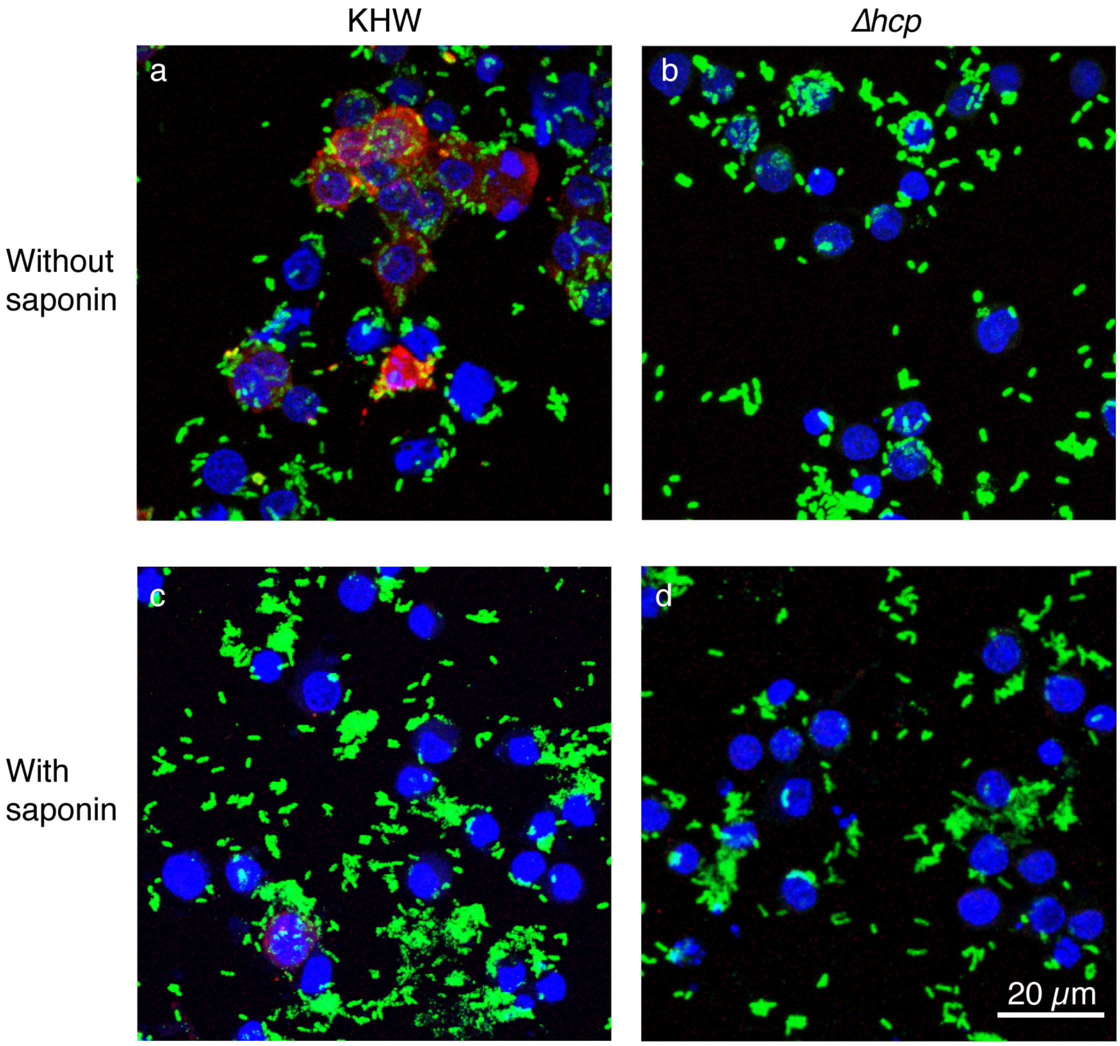

Figure S2: Staining of Hcp with the anti-Hcp1 antibody clone 56-2-2 is incompatible with saponin treatment. Wildtype (KHW)-infected cells stained without (a) or with saponin (c), or Δ*hcp1*-infected cells stained without (b) or with saponin (d). Mammalian nuclei (blue), Hcp (red), bacteria LPS (green).

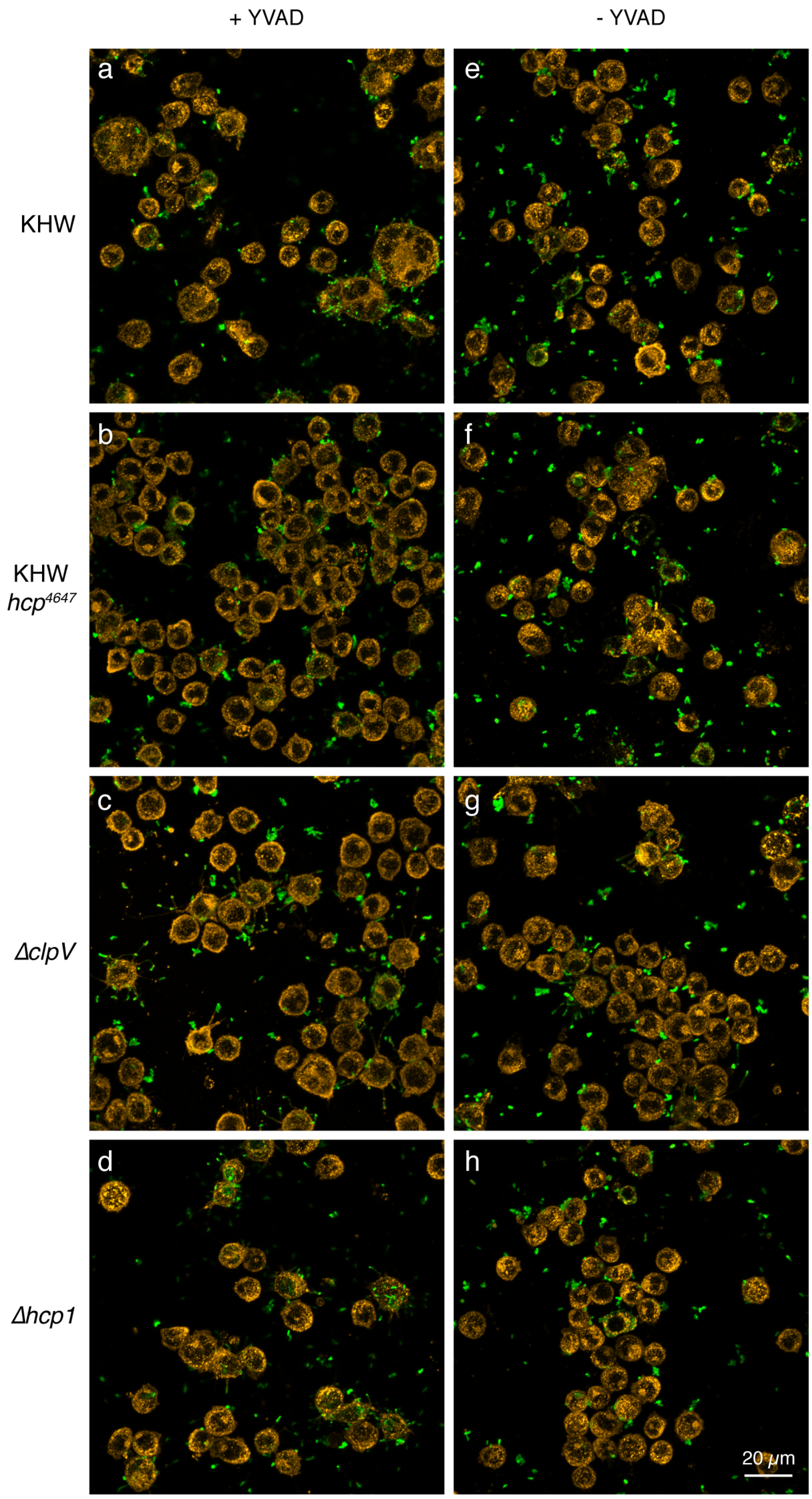

Figure S3: Staining intracellular bacteria in permeabilized infected U937 cells. Fixed infected U937 cells were permeabilized with saponin (a-h). WGA (orange), bacterial LPS (green). This panel is done in duplicate with the panel in Figure 4, except that the cells in this duplicate panel were permeabilized.

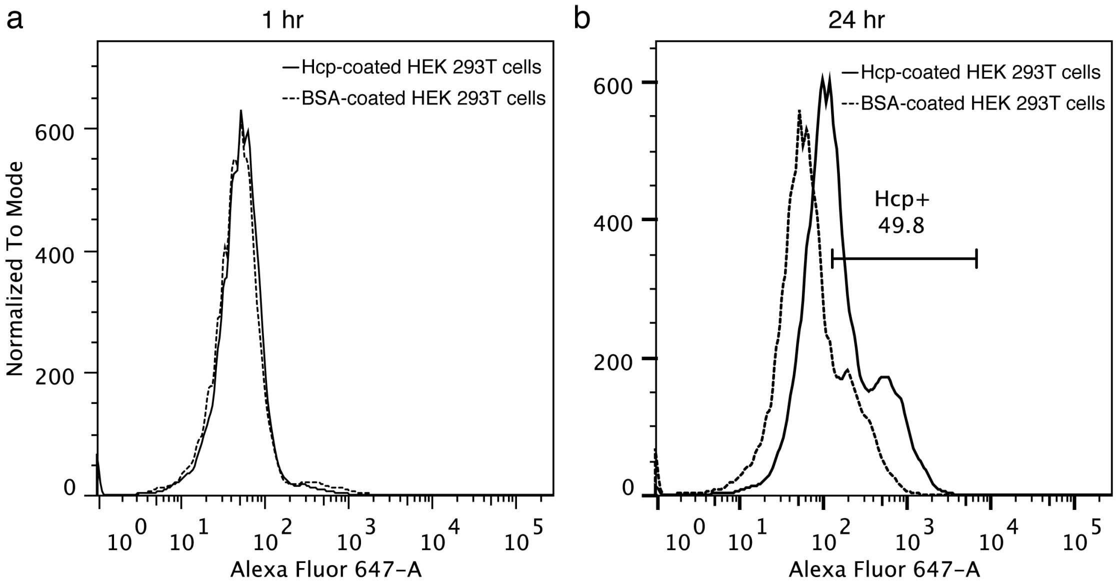

Figure S4: Affinity of Hcp1 for non-immune cells. Hcp1 (solid line) or BSA (dotted line) was incubated with HEK 293T for an hour (a) or 24 hr (b). They were stained with the anti-Hcp monoclonal antibody, followed by anti-mouse IgG AF647 secondary antibody.

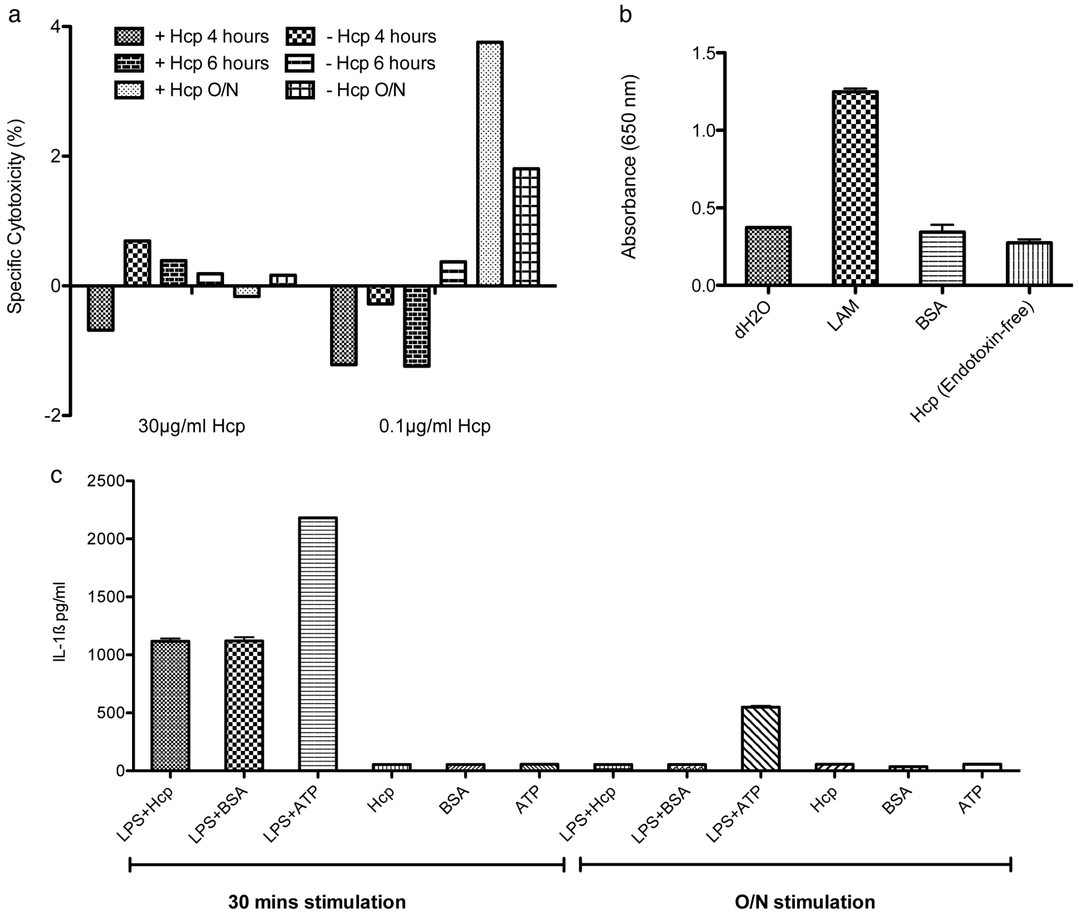

Figure S5: Functional assays on Hcp1. Recombinant Hcp1 was not cytotoxic (a). U937 cells pulsed with chromium-51 were incubated with Hcp1 at indicated time points. Cell lysis was expressed as percentage specific cytotoxicity (%). Hcp1 did not activate NF-κB (b). THP1-Blue cells were treated with LAM, BSA or endotoxin-free rHcp1. The cells were assayed for secreted alkaline phosphatase (absorbance 650 nm), which was the reporter protein for NF-κB activation. Results were expressed as relative light units (R.L.U). Hcp1 did not induce IL-1ß expression (c). J774.1 mouse macrophages were stimulated with combinations of Hcp, BSA, ATP and LPS, for 30 mins or overnight. Levels of IL-1ß in the supernatant were measured and expressed as pg/mL. O/N, overnight; BSA, bovine serum albumin; LAM, lipoarabinomannan.

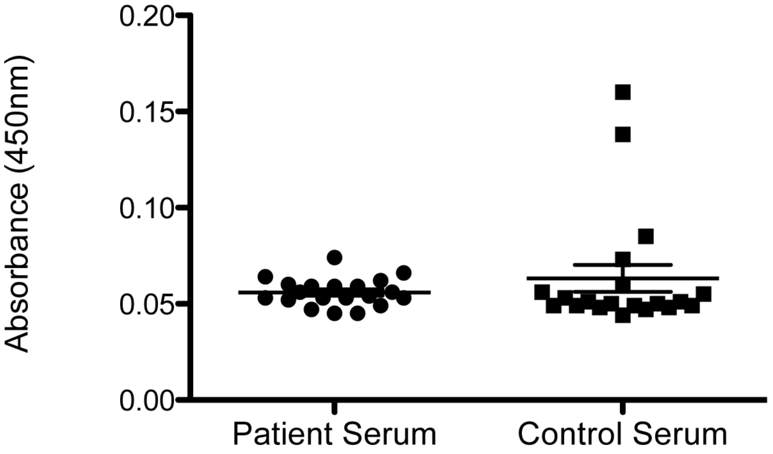

Figure S6: Hcp1 levels in patients' versus controls' sera. Sera were assayed with sandwich ELISA. Results were expressed by absorbance measured at 450nm.

Table S1. Summary of DLS results on wildtype Hcp1 and $Hcp1^{Q46AE47A}$

Sample	Radius (nm)[a]	Polyd (nm)[b]	MW[c]	Sos error[d]
A (Hcp1 2 mg/mL)	5.08	1.15	151	4.62
B (Hcp1 8 mg/mL)	6.50	2.97	270	28.6
C ($Hcp1^{Q46AE47A}$ 2 mg/mL)	4.61	0.99	120	2.94
D ($Hcp1^{Q46AE47A}$ 8 mg/mL)	4.95	1.31	142	7.21

[a]Radius – hydrodynamic radius of the molecule. [b]Polyd – polydispersity parameter. [c]MW – estimated molecular weight. [d]Sos error – value lesser than 50 indicates a good fit.

Table S2. List of primers for this study

Realtime Primers

**Gene**	**Sequences (5'-3')**
hcp1	CACATCCTCGCCTTCAA<br>TCTCGAACTCTTCCATCATCT
rpoB	GTTCCATCGTTCACCAAGTG<br>TTGCAGAAATGTGCTGAATG

Primers for plasmid construction

**Primer Name**	**Sequences (5'-3')**	**PCR Amplification**
Hcp1UpF	CCATGATTACGAATTCGTACGTC GTCGACATGGACA	Forward primer for upstream of *hcp1*
Hcp1UpR	TACCCGGGGATCCTCGATGTGGA TTTTCCCGTCAT	Reverse primer for upstream *hcp1*
Hcp1DnF	GAGGATCCCCGGGTATCACGTTG ACGAAGGAAATG	Forward primer for downstream of *hcp1*
Hcp1DnR	CCAAGCTTGCATGCCTGCAGCGA TCTGCGCTTCGATTT	Reverse primer for downstream of *hcp1*
ClpV UpF	CGGTACCCGGGGATCCGCACTTC GCGTATTTCCA	Forward primer for upstream of *clpV*
ClpV UpR	CGATCAACGGTTTCAGGTC	Reverse primer for upstream *clpV*
ClpV DnF	TGAAACCGTTGATCGCGTCCGAT GCGTCTGAT	Forward primer for downstream of *clpV*
ClpV DnR	GGCCAGTGCCAAGCTTCCCTTCG TCCTTCGTGT	Reverse primer for downstream of *clpV*
$Hcp1^{Q46AE47A}F$	GCGGCGGGCCTGACGCCCGCCG CCGCCGCTCGC	Forward primer for generating $hcp1^{Q46AE47A}$
$Hcp1^{Q46AE47A}R$	CGTCAGGCCCGCCGCGAGCCTGG CAGGCATGTC	Reverse primer for generating $hcp1^{Q46AE47A}$

References:

51. Kohler, G. & Milstein, C. Continuous cultures of fused cells secreting antibody of predefined specificity. 1975. *Biotechnology* **24**, 524-6 (1992).
